# Phytochemical Characterization and Bioactive Properties of Cinnamon Basil (*Ocimum basilicum* cv. ‘Cinnamon’) and Lemon Basil (*Ocimum* *×* *citriodorum*)

**DOI:** 10.3390/antiox9050369

**Published:** 2020-04-29

**Authors:** Chaimae Majdi, Carla Pereira, Maria Inês Dias, Ricardo C. Calhelha, Maria José Alves, Boutayna Rhourri-Frih, Zoubida Charrouf, Lillian Barros, Joana S. Amaral, Isabel C.F.R. Ferreira

**Affiliations:** 1 Centro de Investigação de Montanha (CIMO), Instituto Politécnico de Bragança, Campus de Santa Apolónia, 5300-253 Bragança, Portugal; cmajdiy@gmail.com (C.M.); carlap@ipb.pt (C.P.); maria.ines@ipb.pt (M.I.D.); calhelha@ipb.pt (R.C.C.); maria.alves@ipb.pt (M.J.A.); iferreira@ipb.pt (I.C.F.R.F.); 2 CBMN-UMR 5248, Faculty of Pharmacy, University of Bordeaux, 33016 Bordeaux, France; boutayna.frih@u-bordeaux.fr; 3 Faculté des Sciences, Université Mohammed V, Rabat 10100, Morocco; zcharrouf@yahoo.fr; 4 REQUIMTE/LAQV, Faculdade de Farmácia da Universidade do Porto, 4050-313 Porto, Portugal

**Keywords:** sweet basil, lemon basil, phenolic compounds, volatile compounds, antioxidant activity, antimicrobial activity, anti-proliferative activity

## Abstract

The aim of this work was to contribute to the knowledge on the chemical composition and bioactive properties of two species of the *Ocimum* genus, namely *O. basilicum* cultivar ’Cinammon’ and *O. × citriodorum*. For this purpose, samples of these plants grown in Portugal were evaluated for their composition in phenolic and volatile compounds, and the infusion and hydroethanolic extracts were assessed for their in vitro antioxidant, antimicrobial, cytotoxic, and anti-inflammatory activities. In total, the two basil samples showed the presence of seven caffeic acid and derivatives (dimers, trimers, and tetramers) and five flavonoids, mainly glycoside derivatives of quercetin. Despite some qualitative and quantitative differences, in both samples rosmarinic acid was the major phenolic compound, and linalool the predominant volatile compound. In general, the tested extracts provided relevant bioactive properties since both basil species showed higher antioxidant activity in Thiobarbituric Acid Reactive Substances (TBARs) and Oxidative Hemolysis Inhibition (OxHLIA) assays when compared with the positive control Trolox. Despite *O. × citriodorum* extracts showing slightly better activity against some strains, both types of extracts evidenced similar antimicrobial activity, being more active against Gram-positive bacteria. The extracts also revealed interesting cytotoxicity, particularly the *O. × citriodorum* hydroethanolic extract which was also the only one exhibiting anti-inflammatory activity.

## 1. Introduction

Many species belonging to Lamiaceae family have a long history of culinary use as aromatic herbs or spices, but also in folk medicine. Among those, several belong to the *Ocimum* genus, which is collectively designated as basil and includes more than 30 different species [1]. In the Mediterranean region, one of the most important and frequently consumed species is *Ocimum basilicum*, commonly known as sweet basil or common basil. It is a typical seasoning in many countries, with fresh leaves being consumed in large quantities as an ingredient in several dishes and food preparations. In addition, *O. basilicum* is also cultivated worldwide for its essential oil, with applications in medicine/pharmaceutical, perfume and cosmetics, and as flavoring agent. Moreover, its use as traditional medicinal herb has also been reported, mainly for treating headaches, coughs, digestive, and nervous disorders [2]. *O. basilicum* includes several varieties that have been selected and developed over many years for a variety of purposes, with the existence of at least 18 different cultivars being mentioned by the Herb Society of America [3]. Those comprise of culinary basils, such as cultivar ’Genovese’ or ’Italian Large Leaf’, that were selected for their leaf shape, size, aroma and flavor, while cultivars such as ’Purple Ruffles’ were developed to enhance ornamental traits, for example, leaf color [4].

It is known that different cultivars of basil have the genetic ability of generating different chemical compounds and consequently presenting distinct chemical profiles [5]. Nevertheless, most studies so far focused mainly on the volatile compounds profile, showing the existence of a great variety of chemotypes within the same species of the *Ocimum* genus [6]. However, the chemical composition beyond volatiles and also the bioactive properties of the plants’ extracts are scarcely studied for most *O. basilicum* cultivars as well as for several other species of the *Ocimum* genus. In fact, while many studies have been conducted on *O. basilicum*, only few refer to the cultivar *O. basilicum* ’Cinnamon’ or to the natural hybrid *Ocimum × citriodorum* Vis. under study in this work.

*O. basilicum* cv. ’Cinnamon’, also known as Mexican spice basil or cinnamon basil due to its distinctive cinnamon taste and spicy aroma, is frequently consumed as an ingredient in infusions and baked goods but also in raw dishes, fruit salads and jellies as alternative of ground cinnamon [3,6]. Besides culinary, the plant is also grown for its essential oil, which is used in perfumeries [6]. *O.* × *citriodorum*, popularly known as lemon basil, is a natural hybrid between sweet basil (*O. basilicum*) and African basil (*Ocimum americanum*). This aromatic plant is frequently used both in the Mediterranean and Asian cuisines due to its lemony scent and flavor. In addition, lemon basil has also been used as raw material for the chemical, pharmaceutical and food industries, increasing its economic interest and prompting its cultivation [7].

Despite the interest and wide use of both species, as mentioned, there is still a scarcity of information regarding both plants. Thus, this work aims at filling this gap by evaluating the phytochemical composition of the two plant species, their phenolic compounds and volatiles profile, and also a screening of their biological properties, namely antioxidant, antimicrobial, cytotoxic, and anti-inflammatory activities.

## 2. Materials and Methods

### 2.1. Plant Material and Preparation of the Extracts

Commercial samples (bags of 50 g) of fragmented dried leaves of “cinnamon basil” (*O. basilicum* cv. ’Cinnamon’) and of “lemon basil” (*Ocimum × citriodorum*), were provided by a Portuguese company (Cantinho das Aromáticas, Vila Nova de Gaia, Portugal) dedicated to the production and commercialization of dry aromatic herbs, produced in the Northern region of Portugal under organic farming. Each sample was reduced to a fine powder and stored from light until further analysis.

Two solvents, namely water and ethanol/water (80:20, *v*/*v*), were used to prepare the extracts. To prepare the infusion, 200 mL of boiled distilled water was added to each sample (600 mg) and kept for resting at room temperature for 5 min, followed by filtration, freezing, and lyophilization (FreeZone 4.5, Labconco, Kansas City, MO, USA).

For the hydroethanolic extract preparation, each sample (300 mg) was extracted by stirring with 100 mL of ethanol/water (80:20 *v*/*v*, at 25 °C at 150 rpm) for 1 h and subsequently filtered using Whatman no.4 filter paper (Sigma-Aldrich, St. Louis, MO, USA). The residue was then re-extracted using the same conditions. The combined extracts were evaporated at 35 °C under reduced pressure (rotary evaporator Büchi 3000 series, Buchi AG, Flawil, Switzerland) until the complete removal of ethanol, and afterwards the aqueous phase was frozen and lyophilized.

Additionally, the two basil samples were submitted to essential oil extraction by hydrodistillation in a Clevenger apparatus. For that purpose, approximately 40 g of freshly ground leaves were introduced in a round-bottom flask with 400 mL of distilled water and the mixture was boiled for three hours. After this period, the essential oil was separated from the water and, because a low yield was obtained, the oil was recovered with the addition of 1 mL of hexane. After being collected, the oil was dried over anhydrous sodium sulphate and stored at −20 °C until being analyzed. 

### 2.2. Chemical Composition

#### 2.2.1. Phenolic Compounds

The qualitative and quantitative analysis of the phenolic profile was performed by a liquid chromatography system, Dionex Ultimate 3000 UPLC (Thermo Scientific, San Jose, CA, USA), coupled with a diode-array in series with a Linear Ion Trap mass spectrometry detector equipped with an electrospray ionization source (LC-DAD-ESI/MSn) operating under the conditions previously described by Bessada et al. (2016) [8]. 

The chromatographic separation was carried out on a thermostatted (35 °C) Spherisorb S3 ODS-2 C18 column (3 μm, 4.6 × 150 mm, Waters, Milford, MA, EUA), using the mobile phase conditions (solvents, gradient, and flow rate) described by Bessada et al. (2016) [8]. 

The spectral data for all peaks were recorded at 280 and 370 nm as preferred wavelengths. 

The phenolic compounds were identified by comparing their retention times, UV-vis and mass spectra with those obtained with commercial standard compounds, when available. Otherwise, compounds were tentatively identified comparing the obtained information with available data reported in the literature. For quantitative analysis, a 7-level calibration curve for each available phenolic standard (caffeic acid, rosmarinic acid, quercetin-3-*O*-rutinoside, and quercetin-3-*O*-glucoside, Extrasynthese, Genay, France) was constructed based on the UV signal. For the identified phenolic compounds for which a commercial standard was not available, the quantification was performed through the calibration curve of another compound from the same phenolic group. Each extract was analyzed in triplicate and the results were expressed as mg per g of extract.

#### 2.2.2. Volatile Compounds

The essential oil was analyzed by gas chromatography coupled to mass spectrometry detection (GC-MS) using a GC-2010 Plus (Shimadzu, Kyoto, Japan) system equipped with a AOC-20iPlus (Shimadzu, Kyoto, Japan) automatic injector and a SH-RXi-5ms (30 m × 0.25 mm × 0.25 μm; Shimadzu, Kyoto, Japan, USA) column. The conditions regarding the temperature set for the injector, oven, transfer line, and ion source temperatures were the same as previously described by Spréa et al. (2020) [9]. The volume of the injected sample was 1 μL with a split ratio of 1:10. Helium was used as the carrier gas, adjusted to a linear velocity of 30 cm/s. The ionization energy was 70 eV, scan range was 35–500 u, with a scan time of 0.3 s. The compounds’ identification was based on a comparison of the obtained mass spectra with those of the Nacional Institute of Standards and Technology (NIST2017) mass spectra library and linear retention index (LRI), calculated using the retention times of an n-alkane series (C8-C40, ref. 40147-U, Supelco), and analyzed under identical conditions. The calculated LRI was compared with previously published data [10]. Compounds were quantified as a relative percentage of total volatiles using the relative area values directly obtained from peak total ion current (TIC). Analyses were performed in triplicate.

### 2.3. Bioactive Properties

#### 2.3.1. Evaluation of In Vitro Antioxidant Properties

##### Thiobarbituric Acid Reactive Substances (TBARS)

The TBARS assay was performed following a procedure previously described by Pinela et al. (2012) [11]. Briefly, porcine brain homogenates were prepared and added to a mixture with 0.2 mL of samples extracts, 0.1 mL of FeSO_4_ (0.01mM), and 0.1 mL of ascorbic acid (0.1 mM). The test tubes were incubated at 37 °C for one hour and, after that, 0.5-mL trichloroacetic acid (28% *w*/*v*) was added to stop the reaction. To visualize the extent of oxidation, 0.38 mL of thiobarbituric acid (2%, *w*/*v*) was added and the mixture was heated at 80 °C for 20 min. After the formation of the pink thiobarbituric acid-malondialdehyde complex (TBA-MDA), the tubes were centrifuged at 14,000 rpm for five minutes to remove the precipitated protein and the color intensity of the complex was measured at 532 nm. The inhibition ratio (%) was calculated using the formula,

Inhibition ratio (%) = [(A − B)/A] × 100(1)

where A and B were the absorbance of the control and the extract solution, respectively. Trolox was used as a positive control (in concentrations ranging from 25 to 500 µg/mL). The results were expressed as IC_50_ values (extract concentration able to provide 50% of antioxidant activity, µg/mL).

##### Oxidative Hemolysis Inhibition Assay (OxHLIA)

The OxHLIA assay was performed as described by Lockowandt et al. (2019) [12]. Briefly, 200 μL of a freshly prepared sheep’s erythrocyte suspension (2.8% in PBS, *v*/*v*) was mixed with either 400 μL of extracts dissolved in PBS, water (for complete hemolysis), or PBS solution (as control), using flat-bottom, 48-well microplates. After incubating the plates at 37 °C for 10 minutes while being shaken, 200 μL (160 mM) of the oxidizer 2, 2’-Azobis(2-amidinopropane) dihydrochloride (AAPH) was added to each well. The plate was again incubated at 37 °C with agitation and the optical density was measured at 690 nm every 10 minutes. The following equation was used to evaluate the erythrocyte population that remained intact (P):

P (%) = (St − CH0)/(S0 − CH0) × 100(2)

where S0 and St are the optical density of the sample at 0 and t min, respectively, and CH0 is the optical density of the complete hemolysis at zero minutes. The delayed time of hemolysis (∆t) is calculated as follows:

∆t (min) = Ht50 (sample) − Ht50 (control)(3)

where Ht50 corresponds to the 50% hemolytic time (min) graphically obtained from the hemolysis curve of each antioxidant sample concentration. The Δt values were then linearly correlated to the tested concentrations and from the correlation obtained, the inhibition concentration able to promote a Δt hemolysis delay of 60 min (IC_50_ (60 min), µg/mL) was calculated for each sample. Trolox was used as positive control (in concentrations from 12.5 to 400 µg/mL).

#### 2.3.2. Antimicrobial Activity

The antibacterial activity was assessed by using the microdilution broth method coupled to the rapid *p*-iodonitrotetrazolium chloride (INT) colorimetric assay. The antibacterial activity was assessed against clinical isolates from patients hospitalized in various departments of the Local Health Unit of Bragança, Portugal, comprising five Gram-negative bacteria (*Escherichia coli*, *Klebsiella pneumoniae*, *Morganella morganii*, *Pseudomonas aeruginosa*, and *Proteus mirabilis*, isolated from urine and expectoration) and three Gram-positive bacteria (methicillin-resistant *Staphylococcus aureus* (MRSA), *Listeria monocytogenes*, and *Enterococcus faecalis*). Prior to the assay, the extracts were re-dissolved in 250-μL dimethylsulfoxide (DMSO) and 750 μL of medium and water to obtain a stock solution of 20 mg/mL. Afterwards, they were submitted to further dilution (10 mg/mL to 0.625 mg/mL) using culture medium. The minimum inhibitory concentration (MIC) and minimum bactericidal concentration (MBC) were determined as previously described by Pires et al. (2018). Ampicillin (20 mg/mL), Imipenem (1 mg/mL), and Vancomycin (1 mg/mL) were used as positive controls. Muller Hinton Broth added with 5% DMSO inoculated with each bacterium was used as negative control.

#### 2.3.3. Cytotoxic Activity

The antiproliferative activity of the samples was evaluated by using the Sulforhodamine B (SRB) colorimetric assay as previously described by Abreu et al. (2011) [13]. To obtain stock solutions of 8 mg/mL, each of the lyophilized extracts were re-dissolved in water, and further dilutions from 0.4 until 0.06 mg/mL were prepared. The test was performed using the following human tumor cell lines: MCF-7 (breast adenocarcinoma), NCI-H460 (non-small cell lung cancer), HeLa (cervical carcinoma), and HepG2 (hepatocellular carcinoma), obtained from DSMZ (Leibniz-Institut DSMZ-Deutsche Sammlung von Mirkoorganismen und Zellkulturen GmbH, Braunschweig, Germany). To maintain the tumor cells they were kept in a nutritious medium, namely RPMI-1640 (Hyclone, Logan, USA) medium containing 10% heat-inactivated FBS (Fetal bovine serum, Hyclone, Logan, UT, USA) and 2mM glutamine for MCF-7, NCI-H460 and HCT-15, while HeLa and HepG2 cells were kept in Dulbecco’s Modified Eagle Medium (DMEM, Hyclone, Logan, UT, USA) supplemented with 10% FBS, 2-mM glutamine and antibiotics (100 U/mL penicillin and 100 mg/mL streptomycin, Hyclone, Logan, UT, USA). Briefly, the cells were incubated at 37 °C in a humidified air incubator containing 5% CO_2_ until reaching a sufficient density, after which they were plated in 96-well plates to a density of 1.0×10^4^ cells/well. The cells were left to attach for 24 h and, then, the different sample concentrations were added, and the mixture incubated at the same conditions for 48 h. After that, 100 μL of cold 10% trichloroacetic acid were added followed by another incubation at 4 °C for 60 minutes to fix the cells. After washing with deionized water (4×) and drying, 100 μL of SRB solution (0.1% in 1% acetic acid) was added to each plate well, which was then incubated at room temperature for 30 minutes. After removing the unbound SRB with the addition of 1% acetic acid, the bounded SRB was re-solubilized in 200 μL of 10-mM Tris base solution with agitation for five minutes, and the absorbance was measured at 515 nm using a microplate reader Biotek ELX800 (BioTek Instruments, Inc., Winooski, VT, USA). The results were calculated as GI_50_ values (concentration that inhibits 50% of cell growth). Ellipticine was used as a positive control (in concentrations from 0.3 to 10 μg/mL).

To evaluate the cytotoxicity against non-tumoral cells, namely hepatocytes, an identical procedure was performed using freshly harvested porcine liver cells (PLP2), established as described by Abreu et al. (2011) [13].

#### 2.3.4. Anti-inflammatory Activity

The evaluation of the anti-inflammatory activity was determined as described by Taofiq et al. (2015) [14] using a mouse macrophage-like cell line (RAW264.7) cultured in DMEM medium/HIGH GLUCOSE, at 37 °C under 5% CO2, in humidified air. Briefly, after cells were seeded in the plates, the media was removed, the extracts were added, and plates incubated for 60 min. Subsequently, the cells were stimulated by adding 30 µL of a bacterial lipopolysaccharide solution (10 μg/mL) and culture media to reach a volume of 300 µL. After incubation (24 h, 37 °C, 5% CO_2_, humidified air), the supernatant of each well was transferred to another plate and Griess reagent was added. After 5 min resting at ambient temperature and protected from light, the produced nitric oxide was determined at 515 nm using a microplate reader Biotek ELX800 (BioTek Instruments, Inc., Winooski, VT, USA). Dexamethasone (Sigma-Aldrich, St Louis, MO, USA) was used as positive control (in concentrations from 0.3 to 10 μg/mL). The effect of all the tested samples in the absence of LPS was also evaluated, to observe if they induced changes in nitric oxide basal levels. In negative controls, no LPS was added. The results were calculated by comparison with the standard calibration curve obtained with nitrite solution and expressed as EC_50_.

### 2.4. Statistical Analysis

The results were expressed as mean ± standard deviation (SD) of triplicate analysis. The differences between the different extracts were analyzed using one-way analysis of variance (ANOVA) followed by Tukey’s honestly significant difference post hoc test with α = 0.05, coupled with Welch’s statistic. Differences among extractions and samples were assessed by applying the Student’s *t*-test at a 5% significance level using the SPSS Statistics software (IBM SPSS Statistics for Windows, v. 23.0, IBM Corp., Armonk, NY, USA).

## 3. Results and Discussion

### 3.1. Chemical Composition

#### 3.1.1. Phenolic Compounds Profile

The characteristic data (retention times, λmax, pseudomolecular ion, main fragment ions in MS2) of the phenolic compounds identified in the prepared extracts are listed in Table 1. 

In total, the two basil samples showed the presence of seven caffeic acid and derivatives (dimers, trimers and tetramers of rosmarinic acid) and five flavonoids, mainly glycoside derivatives of quercetin. Caffeic acid (compound 2), quercetin-3-*O*-rutinoside (compound 5), quercetin-3-*O*-glucoside (compound 7), and rosmarinic acid (compound 10) were positively identified according to their retention, mass spectra and UV-vis characteristics in comparison with commercial standards. Compounds 1 ([M − H]^−^ at *m/z* 341) and 4 ([M − H]^−^ at *m/z* 473) presented a fragmentation pattern that allowed assigning them as caffeic acid hexoside and chicoric acid (dicaffeoyltartaric acid), respectively. This last compound, as well as compounds 2 and 10 (caffeic acid and rosmarinic acid, respectively), have been described by several authors as being the main phenolic acids in basil [15,16,17,18,19,20]. Compounds 6 and 11 ([M − H]^−^ at *m/z* 717) presented a fragmentation pattern with successive losses of 198 u (danshensu) or 180 u (caffeic acid) units, coherent with salvianolic acid B (also known as lithospermic acid B) [21,22]. Compound 12 ([M − H]^−^ at *m/z* 537) presented a UV spectrum and fragmentation pattern consistent with the caffeic acid trimer lithospermic acid A. Salvianolic acids H/I, with the same molecular weight as lithospermic acid A, were discarded as possible identities because they present quite a different fragmentation pattern [22,23]. Furthermore, the presence of lithospermic acid A in an unnamed sweet basil cultivar was already reported by Lee & Scagel [16]. Based on the obtained data, the remaining compounds (peaks 4, 8, and 9) were assigned to quercetin glycosides derivatives. Compound 4 ([M − H]^−^ at *m/z* 595) released two MS^2^ fragments at *m/z* 463 ([M − H-132]^−^ and 301 ([M − H-162]^−^, which revealed the alternative loss of a pentosyl and hexosyl moieties, being tentatively identified as quercetin-*O*-pentoside-*O*-hexoside. Meanwhile, compounds 8 and 9 ([M − H]^−^ at *m/z* 549) presented three MS^2^ fragments at *m/z* 505, 463, and 301 (quercetin; [M − H-44-42-162]^−^, loss of an malonyl-hexoside moiety), being assigned as quercetin-*O*-malonyl-hexoside.

Comparing the two basil samples under study, a higher content of total phenolic compounds (TPC) was obtained for the hydroethanolic extract of cinnamon basil, while for lemon basil higher amounts were obtained for the infusion. Lithospermic acid A was only identified in *O. basilicum* cv. ’Cinnamon’ while, interestingly, different Salvianolic acid B isomers were found in each basil species. In both samples, irrespective of the extraction method, rosmarinic acid was the main phenolic compound, which is in good agreement with previous works that reported this phenolic acid as being the main compound present in *O. basilicum* [16,24,25,26]. Nevertheless, most of the previous works on the phenolic composition of *O. basilicum* samples do not mention the cultivar used or refer to commercial samples, also without specifying the cultivar. In the study performed by Simeoni et al. (2018) [27] using a commercial *O. basilicum* sample (non-identified cultivar), 13 phenolic acids and three flavonoids were identified and quantified, among which chicoric acid was the major compound, followed by rosmarinic acid. The major flavonoid was isoquercetin, while in the present work it was found to be quercetin-3-*O*-rutinoside. Different flavonoids, such as kaempferol, apigenin, and luteolin derivatives, and phenolic acids, such as chlorogenic acid, were reported by Jayasinghe et al. (2003) [15] in the fraction of a methanolic extract prepared with *O. basilicum* grown in Sri Lanka, but again the cultivar was not discriminated, which can possibly explain, at least partially, the different profiles. Similarly, Hossain et al. (2010) [17] identified a total of 33 compounds in a basil hydroethanolic (80%) extract, of which 24 were described for the first time, despite no quantitative data being given. The identified compounds included several flavonoids and phenolic acids distinct of the ones found in the present study. However, the authors only mentioned that it was a commercial basil spice sample bought from Turkey, without even referring to the species used. As far as the literature consulted on the phenolic composition of sweet basil, only one previous study was found that included the cultivar ’Cinnamon’ [18]. The study included 15 different cultivars of *O. basilicum*; nevertheless, only the four major phenolic acids were evaluated. The ’Cinnamon’ cultivar presented rosmarinic acid as the main phenolic acid, followed by chicoric acid, which is in good agreement with the present results. Nevertheless, the third major compound was caftaric acid, which was not detected in the present study. Despite the phenolic acids content reported by Kwee and Niemeyer (2011) [18] in *O. basilicum* cultivar ’Cinnamon’ being much lower than the ones presented on Table 1, these values are not comparable because the results are differently expressed. 

In what concerns *O. ×* citriodurum phenolic composition, data from the literature was also found to be very scarce. Hakkim et al. (2008) [28] evaluated the phenolic composition, using HPLC-DAD, of the methanolic extracts of eight different *Ocimum* species, including *O. ×* citriodurum. The authors reported the presence of seven phenolic acids, namely rosmarinic, lithospermic, vanillic, p-coumaric, hydroxybenzoic, ferulic, and cinnamic acids, in *O. ×* citriodurum. Contrary to the results obtained in the present work (Table 1), hydroxybenzoic (0.20 mg/g dry extract) and p-coumaric (0.19 mg/g dry extract) were found to be the main compounds. In addition, in the present study, the contents of rosmarinic and lithospermic acids were much higher than those reported by Hakkim et al. (2008) [28].

#### 3.1.2. Volatile Compounds

Table 2 lists the compounds identified by GC-MS in the essential oil of cinnamon basil and lemon basil. A total of 59 and 75 compounds were detected for *O. basilicum* cultivar ’Cinnamon’ and *O. × citriodorum*, respectively, representing 99.2% and 88.3% of the total compounds in each essential oil (Table 2). Despite the qualitative and quantitative differences between the composition of the two oils, linalool was found to be the major compound in both samples. In cinnamon basil, it was followed by (E)-methyl-cinnamate, which presented also a very high amount (24.7%), and τ-cadinol (7.4%), while in lemon basil by caryophyllene oxide (6.2%) and trans-α-bergamotene (5.7%). Considering relative percentages, lemon basil presented a higher content of oxygenated monoterpenes and of sesquiterpenes whereas cinnamon basil was rich in the cinnamic acid derivative methyl-cinnamate, which was absent in the former essential oil, and presented slightly higher amounts of oxygenated sesquiterpenes and monoterpenes. When compared to previously reported data, the profile of *O. basilicum* cultivar ’Cinnamon’ was very similar to that described by Wesolowska and Jadczak (2016) [6] regarding the same cultivar cultivated in North-western Poland, but completely distinct to that reported by Tsasi et al. (2017) [29]. The chemical profile of *O. basilicum* cultivar ’Cinnamon’ samples collected in the Island of Kefalonia, Greece, showed methyl chavicol as major compound (ranging from 60.2–75.1%) followed by linalool (0.6–5.7%) and germacrene D (3.2–5.0%), while in the present study the content of methyl chavicol was much lower (2.5%). According to Vieira and Simon (2006) [4] who studied the volatile profile of different basil species, *O. basilicum* cv. ’Cinnamon’ can be differentiated from three other *Ocimum* species (*O.* americanum, *O. × citriodorum* and *O.* minimum) and 14 *O. basilicum* cultivars by the high amount of (*E*)-methyl-cinnamate, which is in good agreement with the data reported by Wesolowska and Jadczak (2016) [6] and the present study.

The volatile profile obtained for the *O. × citriodorum* sample showed striking differences when compared to other previous studies. Vieira and Simon (2006) [4] analyzed the essential oil from four accessions of *O. × citriodorum* grown in Purdue, USA, together with several *O. basilicum* cultivars, and reported the existence of two types of *O. × citriodorum*, one rich in citral isomers (neral + geranial) and another rich in methyl-chavicol, with three accessions belonging to the first type and one to the second. Despite presenting qualitative and quantitative differences, other studies supported the citral-rich type as the most commonly observed chemotype of *O. × citriodorum* [5,7,30,31], although other studies also confirmed the existence of methyl-chavicol-rich type [32]. Nevertheless, the volatile profile of the present studied sample does not fit either type as it presented linalool as major compound (32.8%) and citral being only the fifth major compound, representing 4.9% of total volatiles. Curiously, the obtained profile is much closer to the one reported by Vieira and Simon (2006) [4] for *O. basilicum* cultivar ’Mrs. Burns’ Lemon’, which is also a basil plant with a strong lemon aroma [3].

### 3.2. Bioactive Properties

#### 3.2.1. Antioxidant Activity

Table 3 shows the results obtained for the TBARS and OxHLIA assays. In what concerns the former assay, in general, the extracts of the two studied species were more efficient in inhibiting the formation of TBARS (resulting from the oxidation of the brain cell membranes, with consequent formation of malondialdehyde) when compared to the synthetic antioxidant Trolox, a water-soluble analogue of vitamin E. The IC_50_ of the extracts were significantly lower than that of Trolox, in a magnitude of approximately 10x for almost all extracts. This suggest a high efficacy in inhibiting the formation of TBARS, particularly for the infusions that showed the lowest IC_50_ values (8.9 ± 0.4 µg/mL for cinnamon basil and 14.1 ± 0.7 µg/mL for lemon basil).

In the OxHLIA assay, in general, the Δt values were well correlated to the tested extract concentrations, since higher concentrations promoted a higher hemolysis delay. Trolox, used as positive control, presented an IC_50_ value of 85 ± 2 μg/mL, while the IC50 values for all the tested basil extracts were much lower. Additionally, in this assay, the infusions performed better than the hydroethanolic extracts, with the former requiring approximately half the concentration of the last to protect 50% of the erythrocyte population from the haemolytic action of the oxidative agent after 60 min. These results suggest that both the studied species have high antioxidant properties, therefore presenting a noteworthy potential to confer beneficial health effects when consumed in the infusion form.

To the best of our knowledge, there are no previous reports on the antioxidant activity of the infusions of these plants, assessed through cell-based assays and using Trolox as positive control. Nevertheless, previous studies have been conducted on the ability of the ethanolic extracts of *O. basilicum* cv. ’Cinnamon’ and methanolic extracts of *O. × citriodorum* to act as antioxidants. Contrary to the results obtained in the present study, the extract of *O. basilicum* presented a lower antioxidant activity than t-butylhydroxytoluene (BHA) in the screening assay of 2,2-diphenyl-1-picrylhydrazyl (DPPH) radicals scavenge (Abramovič et al., 2018), but the results are not directly comparable to the ones obtained in the present study because they are expressed as percentage of antioxidant activity and no IC_50_ values are provided. Additionally, Hakkim et al. (2008) [28] found that the extract of *O. × citriodorum* tend to possess lower antioxidant activity than the positive control butylated hydroxy anisole (BHA) in DPPH, reducing power, superoxide anion scavenging activity and β-carotene-linoleic acid bleaching assays. On the other hand, in the study of Kaurinovic et al. (2011) [33], the aqueous extract of a non-identified cultivar of *O. basilicum* showed better antioxidant activity compared to BHT and BHA in the DPPH assay, and better activity than BHT in the neutralization of NO radical and H_2_O_2_. Additionally, the extracts were able to inhibit the lipid peroxidation in liposomes, with the largest inhibitory activity being exhibited by the ethyl acetate extract. Likewise, Touiss et al. (2019) [34] reported that a rosmarinic acid-rich extract prepared from a commercial sample of *O. basilicum* (non-identified cultivar) was able to significantly decrease the plasma total cholesterol, triglycerides, and LDL-cholesterol in high fat diet-induced hyperlipidemic mice and also prevent lipoprotein oxidation by 93% at a dose of 25µg/ml. These results are in accordance with the ones obtained in the present study, which corroborate a strong antioxidant activity for the studied *Ocimum* plant species.

#### 3.2.2. Antimicrobial Activity

The results obtained for the antimicrobial activity of the two types of extracts prepared from *O. basilicum* and *O. × citriodorum* can be observed on Table 4. The results evidenced that all extracts present antibacterial activity since they were able to inhibit the growth of all tested strains, with the exception of P. mirabilis. Nevertheless, none of the extracts showed bactericidal activity at the tested concentrations. In general, better results were obtained for Gram-positive bacteria, with the lowest MIC (5 mg/mL) being observed for MRSA, a pathogenic nosocomial bacterium. These results are in line with previous studies on the antimicrobial activity of hydroethanolic extracts obtained from other plant species [35]. Observing the obtained MIC values (Table 4), one can conclude that the antimicrobial activity between the two types of extracts is very similar against most of the assayed strains, with the exception of MRSA for which lower MICs were obtained for the hydroethanolic extract. Better results were also obtained for the hydroethanolic extract of cinnamon basil against *L. monocytogenes* and for the hydroethanolic extract of lemon basil for against *P. aeruginosa*. Notwithstanding, the two basil samples showing similar results for most of the tested bacteria, slightly better activity was evidenced by the extracts of *O. × citriodorum*, in particular against the Gram-negative *E. coli* and *K. pneumoniae*. The antimicrobial activity evidenced by the extracts may be related to the presence of rosmarinic acid as major compound in these extracts since previous studies reported several biological properties associated to this phenolic acid, including antibacterial activity [36].

#### 3.2.3. Cytotoxic Activity and Anti-inflammatory Activity

The results for the anti-proliferative activity, hepatotoxicity, and anti-inflammatory activity of the aqueous and hydroethanolic extracts of the studied basil samples are shown in Table 5. The results are expressed in terms of GI_50_ values corresponding to sample concentration providing 50% of cell growth inhibition. As can be observed, the hydroethanolic extract of *O. × citriodorum* was the only that showed cytotoxicity against the four human tumor cell lines used with GI_50_ values ranging from 89 to 161 µg/mL. This was also the only extract that presented anti-inflammatory activity in the mouse macrophage-like cell line (RAW264.7) assay (Table 5). Nevertheless, it also exhibited cytotoxicity towards non-tumoral hepatocytes, despite presenting a higher GI_50_ value (234 ± 21 µg/mL) compared to the one obtained for cancer cell lines. Regarding the three other extracts, all of them were able to inhibit the growth of all tumoral cells with exception of non-small cell lung cancer (NCI-H460 cell line), without exhibiting cytotoxicity for non-tumor cells at the tested concentrations. 

Recently, Qamar et al. (2020) [37] screened the activity of *O. basilicum* (aerial parts non-identified cultivar) methanolic extract and fractions against several human cancer cell lines (HT-144, MCF-7, NCI-H460 and SF-268) using the same methodology as the one in this work. The authors reported that both the methanolic extract and the petroleum ether insoluble fraction showed growth inhibitory effects against all the four cell lines tested, with both exhibiting a selectively greater inhibition against the MCF-7 cell line. This is in good agreement with the results of the present work, in which MCF-7 cell line also presented the lowest GI50 value.

## 4. Conclusions

Until now, different studies are reported in the literature regarding *Ocimum* species, but few focused specifically on the two studied in this work, *O. basilicum* cv. Cinnamon and *O. × citriodorum*. The obtained results demonstrated that both plants are of great interest, both for their aromatic characteristics and composition in bioactive compounds, particularly of phenolic acids such as rosmarinic acid, but mainly for their bioactive properties. All the extracts presented relevant antioxidant activity with the infusions of both plants evidencing remarkable results in TBARs and OxLIA assays. The extracts of both basil species were able to inhibit different tumor cell lines, with the majority not affecting the normal liver cells. In addition, the hydroethanolic extract of lemon basil (*O. × citriodorum*) showed anti-inflammatory activity. In general, the overall results obtained for *O. basilicum* and *O. × citriodorum* support the use of both species in traditional medicine and confirm the relevance of these plants as a natural source of bioactive compounds both when consumed as foods or infusions.

## Figures and Tables

**Table 1:** Retention time (Rt), wavelengths of maximum absorption in the visible region (λmax), mass spectrometric data, tentative identification, and quantification (mg/g of extract) of the phenolic compounds in *Ocimum basilicum* ’Cinnamon’ and *Ocimum × citriodorum* hydroethanolic extracts and infusion preparations.

Peak	Rt (min)	λmax (nm)	[M − H]^−^ (*m/z*)	MS^2^ (*m/z*)	Tentative Identification	Quantification (mg/g of Extract)			
						*Ocimum basilicum* cv. ’Cinnamon’		*Ocimum × citriodorum*	
						EtOH:H_2_O	Infusion	EtOH:H_2_O	Infusion
**1**	4.81	326	341	179(100),161(62),135(34)	Caffeic acid hexoside ^A^	tr	tr	tr	tr
**2**	9.89	323	179	135(100)	Caffeic acid ^A^	1.194 ± 0.002 ^c^	0.63 ± 0.02 ^d^	1.41 ± 0.03 ^b^	3.1 ± 0.1 ^a^
**3**	13.54	327	473	311(100),293(98),179(8),149(5),135(5)	Chicoric acid ^A^	0.51 ± 0.03 ^d^	0.61 ± 0.01 ^c^	0.64 ± 0.01 ^b^	1.09 ± 0.05 ^a^
**4**	15.88	350	595	463(25),301(100)	Quercetin-*O*-pentoside-*O*-hexoside ^B^	2.07 ± 0.06 ^a^	1.54 ± 0.02 ^b^	nd	nd
**5**	17.63	355	609	301(100)	Quercetin-3-*O*-rutinoside ^B^	4.53 ± 0.01 ^a^	3.24 ± 0.04 ^b^	1.525 ± 0.003 ^d^	2.85 ± 0.02 ^c^
**6**	18.21	340	717	537(40),519(100),493(15),359(10),339(8),321(5)	Salvianolic acid B isomer 1 ^C^	nd	nd	2.23 ± 0.04 ^b^	2.47 ± 0.03 ^a^
**7**	18.53	341	463	301(100)	Quercetin-3-*O*-glucoside ^D^	3.405 ± 0.003 ^a^	2.22 ± 0.04 ^d^	2.91 ± 0.08 ^b^	2.43 ± 0.02 ^c^
**8**	20.05	337	549	505(5),463(28),301(100)	Quercetin-*O*-malonyl-hexoside ^B^	3.4 ± 0.1 ^a^	2.82 ± 0.03 ^b^	1.87 ± 0.02 ^d^	2.11 ± 0.03 ^c^
**9**	20.45	337	549	505(6),463(48),301(100)	Quercetin-*O*-malonyl-hexoside ^B^	nd	nd	1.58 ± 0.02 ^b^	1.84 ± 0.05 ^b^
**10**	21.75	339	359	197(25),179(41),161(100),135(5)	Rosmarinic acid ^C^	77 ± 1 ^a^	41.0 ± 0.2 ^d^	50 ± 1 ^c^	59.1 ± 0.3 ^b^
**11**	25.79	329	717	537(5),519(100),493(5),339(5),321(7),295(5)	Salvianolic acid B isomer 2 ^C^	5.2 ± 0.1 ^c^	7.3 ± 0.4 ^a^	nd	6.9 ± 0.1 ^b^
**12**	30.44	284/329	537	493(100),439(5),359(62),197(5),179(10),161(15)	Lithospermic acid A ^C^	7.11 ± 0.02 ^a^	2.82 ± 0.06 ^b^	nd	nd
					**Total Phenolic Acids**	**91 ± 1 ^a^**	**52.4 ± 0.4 ^d^**	**55 ± 1 ^c^**	**72.7 ± 0.3 ^b^**
					**Total Flavonoids**	**13.4 ± 0.2 ^a^**	**9.8 ± 0.1 ^b^**	**7.9 ± 0.1 ^d^**	**9.2 ± 0.1 ^c^**
					**Total Phenolic Compounds**	**105 ± 1 ^a^**	**62.2 ± 0.1 ^c^**	**63 ± 2 ^c^**	**81.9 ± 0.2 ^b^**

nd—not detected. tr—traces. Standard calibration curves used for compounds’ quantification: ^A^—caffeic acid (*y* = 388345*x* + 406369, *R^2^* = 0.9939, limit of detection (LOD) and limit of quantitation (LOQ) = 0.78 and 1.97 µg/mL, respectively); ^B^—quercetin-3-*O*-rutinoside (*y* = 13343*x* + 7675, *R^2^* = 0.9998, LOD and LOQ = 0.18 and 0.65, respectively); ^C^—rosmarinic acid (*y* = 191291*x* – 652903, *R^2^* = 0.999, LOD and LOQ = 0.15 and 0.68, respectively); ^D^—quercetin-3-*O*-glucoside (*y* = 34843*x* – 160173, *R^2^* = 0.9998, LOD and LOQ = 0.21 and 0.71, respectively). Different letters (lowercase letters) correspond to significant differences (*p* < 0.05).

**Table 2:** Profile of volatile compounds identified by GC-MS in the essential oil of basil samples.

Compound		RT	LRI ^a^	LRI ^b^	Quantification ^c^ (Relative %)	
					*O. basilicum* cv. ’Cinnamon’	*O. × citriodorum*
1	1-Hexanal	7.90	800	801	−	0.014 ± 0.001
2	2-Hexenal	10.23	850	846	−	0.012 ± 0.001
3	α-Thujene	13.90	926	924	0.07 ± 0.003	0.044 ± 0.002
4	α-Pinene	14.22	932	932	0.036 ± 0.001	0.245 ± 0.005
5	Camphene	14.96	947	946	0.0157 ± 0.0004	0.022 ± 0.003
6	Benzaldehyde	15.55	958	952	0.049 ± 0.003	0.012 ± 0.001
7	Sabinene	16.30	972	969	0.019 ± 0.001	0.014 ± 0.003
8	β-Pinene	16.43	975	974	0.064 ± 0.002	0.092 ± 0.002
9	1-Octen-3-ol	16.69	980	974	−	0.138 ± 0.002
10	6-Methyl-5-hepten-2-one	17.05	987	985	−	0.229 ± 0.003
11	β-Myrcene	17.27	991	988	0.045 ± 0.001	0.061 ± 0.002
12	4-carene	18.54	1015	1011	0.038 ± 0.002	0.023 ± 0.005
13	*o*-Cymene	18.95	1023	1022	0.034 ± 0.002	0.252 ± 0.007
14	D-Limonene	19.16	1027	1024	0.055 ± 0.002	−
15	Eucalyptol	19.28	1030	1026	1.16 ± 0.04	−
16	1,8-Cineole	19.33	1031	1026	−	0.02 ± 0.03
17	Benzeneacetaldehyde	19.91	1042	1036	0.079 ± 0.006	0.082 ± 0.005
18	*trans*-Ocimene	20.23	1048	1044	0.066 ± 0.001	0.013 ± 0.002
19	Bergamal	20.51	1053	1051	−	0.029 ± 0.002
20	γ-Terpinene	20.75	1058	1054	0.080 ± 0.004	0.435 ± 0.009
21	*trans*-4-thujanol	21.17	1066	1065	0.045 ± 0.002	0.017 ± 0.004
22	*cis*-Linalool oxide	21.46	1072	1067	0.26 ± 0.01	1.67 ± 0.03
23	*n*-Octanol	21.97	1082	−	−	1.071 ± 0.008
24	*trans*-Linalool oxide	22.27	1088	1084	0.029 ± 0.01	1.73 ± 0.05
25	Rosefuran	22.77	1097	1093	−	0.079 ± 0.005
26	Linalool	23.09	1101	1095	26.5 ± 0.3	32.8 ± 0.4
27	1-Octen-3-yl acetate	23.80	1118	1110	−	0.011 ± 0.002
28	p-Menth-2-en-1-ol	24.27	1127	1124	−	0.011 ± 0.002
29	4-Acetyl-1-methylcyclohexene	24.64	1135	1137	−	0.019 ± 0.002
30	Camphor	25.10	1144	1141	0.321 ± 0.006	0.177 ± 0.003
31	*p*-Menthan-3-one	25.57	1154	1148	0.024 ± 0.005	−
32	Nerol oxide	25.73	1157	1154	−	0.012 ± 0.002
33	Borneol	26.19	1166	1165	0.018 ± 0.009	−
34	Isoneral	26.20	1166	1160	−	0.12 ± 0.01
35	δ-terpineol	26.39	1170	1162	−	0.11 ± 0.02
36	Menthol	26.52	1173	1167	0.06 ± 0.02	−
37	*trans*-Pyranoid linalool oxide	26.54	1173	1173	−	0.079 ± 0.006
38	Terpinen-4-ol	26.75	1177	1174	0.71 ± 0.02	4.96 ± 0.01
39	*p*-Cymen-8-ol	27.11	1185	1179	0.016 ± 0.001	0.056 ± 0.002
40	α-Terpineol	27.40	1190	1186	0.32 ± 0.01	0.628 ± 0.006
41	Dihydrocarveol	27.70	1196	1192	−	0.09 ± 0.01
42	Methyl chavicol	27.79	1198	1195	2.46 ± 0.01	0.285 ± 0.009
43	Octanol acetate	28.40	1211	1211	0.019 ± 0.004	0.132 ± 0.002
44	Fenchyl acetate	28.84	1221	1218	0.028 ± 0.003	−
45	Nerol	29.19	1234	1227	0.036 ± 0.002	3.70 ± 0.05
46	Carvone	29.93	1244	1239	0.0355 ± 0.0001	0.34 ±0.01
47	Neral	29.98	1245	1235	−	2.21 ± 0.04
48	Geraniol	30.60	1258	1249	−	1.60± 0.01
49	Geranial	31.16	1270	1264	0.012 ± 0.001	2.68 ± 0.02
50	Neryl formate	31.71	1282	1280	−	0.054 ± 0.001
51	Bornyl acetate	31.93	1287	1284	0.38 ± 0.01	−
52	Carvacrol	32.69	1303	1298	−	0.202 ± 0.008
53	(*Z*)-Methyl cinnamate	32.83	1306	1299	3.14 ± 0.02	0.054 ± 0.005
54	Methyl geranate	33.64	1324	1322	−	0.078 ± 0.001
55	Myrtenyl acetate	33.72	1326	1324	0.0214 ± 0.0004	−
56	exo-2-Hydroxycineole acetate	34.45	1343		0.037 ± 0.003	−
57	α-Cubebene	34.84	1352	1345	0.157 ± 0.003	0.456 ± 0.003
59	Eugenol	35.16	1359	1356	2.18 ± 0.04	0.323 ± 0.001
60	Neryl acetate	35.46	1366	1359	−	1.04 ± 0.01
61	α-Copaene	36.02	1379	1374	0.40 ± 0.02	1.011 ± 0.005
62	(*E*)-Methyl cinnamate	36.46	1384	1376	24.70 ± 0.06	−
63	β-Bourbonene	36.47	1389	1387	−	0.313 ± 0.002
64	β-Elemene	36.78	1395	1389	3.00 ± 0.05	0.27 ± 0.03
65	*n*-Tetradecane	36.98	1400	1400	−	0.012 ± 0.001
66	cis-α-Bergamotene	37.78	1419	1411	−	0.012 ± 0.001
67	β-Ylangene + β-cedrene	37.93	1423	1419; 149	0.335 ± 0.009	−
68	(E)-Caryophyllene	37.98	1424	1417	−	4.32 ± 0.03
69	β-Copaene	38.37	1434	1430	−	0.120 ± 0.002
70	*trans*-α-Bergamotene	38.57	1438	1432	0.66 ± 0.03	5.76 ± 0.04
71	α-Guaiene	38.72	1442	1437	1.26 ± 0.02	−
72	Muurola-3,5-diene	39.05	1450	1448	0.011 ± 0.001	−
73	Geranyl acetone	39.21	1454	1453	−	0.109 ± 0.002
74	trans-β-farnesene+ humulene	39.44	1459	1454;1542	−	1.14 ± 0.03
75	Sesquisabinene	39.53	1461	1457	−	0.056 ± 0.002
76	γ-Muurolene	40.39	1482	1478	−	0.10 ± 0.01
77	Germacrene D	40.53	1486	1480	3.17 ± 0.03	3.70 ± 0.02
78	β-Selinene	40.75	1491	1489	0.28 ± 0.02	0.585 ± 0.003
79	epi-cubebol + α-Selinene	41.14	1500	1493;1492	−	0.54 ± 0.01
80	α-Bulnesene	41.53	1510	1509	2.26 ± 0.03	−
81	β-Bisabolene	41.58	1511	1505	−	0.49 ± 0.04
82	γ-Cadinene	41.87	1519	1513	3.76 ± 0.03	0.41 ± 0.04
83	δ-Cadinene	42.18	1527	1522	0.63 ± 0.05	−
84	Cadina-1(10),4-diene	42.22	1528	1522	−	0.71 ± 0.03
85	epi-Cubebol	42.49	1534	1533	0.15 ± 0.01	−
86	α-Cadinene	42.77	1541	1537	0.09 ± 0.01	−
87	(E)-Nerolidol	43.68	1565	1561	0.87 ± 0.03	−
88	(Z)-Nerolidol	43.79	1567	1561	−	0.46 ± 0.02
89	Spathulenol	44.38	1583	1577	1.375 ± 0.003	−
90	Caryophyllene oxide	44.61	1589	1582	0.35 ± 0.04	6.235 ± 0.008
91	Salvial-4(14)-en-1-one	45.07	1600	1594	−	0.209 ± 0.006
92	Humulene epoxide II	45.69	1616	1608	−	0.151 ± 0.001
93	1,10-Di-epi-cubenol	45.82	1620	1618	1.33 ± 0.07	−
94	τ-Cadinol	46.80	1646	1638	7.44 ± 0.03	0.521 ± 0.002
95	β-Eudesmol	47.17	1656	1649	0.33 ± 0.01	−
96	α-Cadinol	47.28	1659	1652	0.6 ± 0.2	−
97	α-Bisabolol	48.33	1687	1685	−	0.499 ± 0.004
98	Hexahydrofarnesyl acetone	52.07	1849	1847	0.09 ± 0.01	0.105 ± 0.006
						
	Total identified				92.2 ± 0.2	88.3 ± 0.2
	Monoterpenes				4.6 ± 0.1	3.12 ± 0.06
	Oxygenated monoterpenes				31.8 ± 0.2	55.1 ± 0.3
	Sesquiterpenes				13.1 ± 0.2	20.2 ± 0.1
	Oxygenated sesquiterpenes				12.4 ± 0.2	8.1 ± 0.1
	Other				30.3 ± 0.5	1.79 ± 0.07

^a^ LRI, linear retention index determined on a SH- RXi-5ms fused silica column (Shimadzu) relative to a series of n-alkanes (C8–C40). ^b^ linear retention index reported in literature (Adams, 2017) [10]. ^c^ relative % is given as mean ± SD, *n* = 3.

**Table 3:** Results of the antioxidant activity assays (TBARS and OxHLIA) obtained for the infusion and hydroethanolic extracts of basil samples (mean ± SD, *n* = 3).

Sample		TBARS (IC_50_; µg/mL)	OxHLIA (IC_50_; µg/mL) Δt = 60 min
*O. basilicum* cv. ’Cinnamon’	Infusion	8.9 ± 0.4	27.6 ± 0.9
	EtOH:H_2_O	23.8 ± 0.8	48 ± 2
*O. × citriodorum*	Infusion	14.1 ± 0.7	26.9 ± 0.4
	EtOH:H_2_O	15.6 ± 0.6	54 ± 1
Trolox		139 ± 5	85 ± 2

**Table 4:** Antimicrobial activity of the extracts obtained from the basil samples (mg/mL, mean ± SD, *n* = 3).

Antimicrobial Activity	*O. basilicum* cv. ’Cinnamon’				*O. × citriodorum*				Ampicillin (20 mg/mL)		Imipenem (1 mg/mL)		Vancomycin (1 mg/mL)	
	EtOH/H_2_O		Infusion		EtOH/H_2_O		Infusion							
	MIC	MBC	MIC	MBC	MIC	MBC	MIC	MBC	MIC	MBC	MIC	MBC	MIC	MBC
**Gram-negative bacteria**														
*E. coli*	20	>20	20	>20	10	>20	10	>20	<0.15	<0.15	<0.0078	<0.0078	n.t.	n.t.
*K. pneumoniae*	20	>20	20	>20	10	>20	10	>20	10	20	<0.0078	<0.0078	n.t.	n.t.
*M. morganii*	10	>20	10	>20	10	>20	10	>20	20	>20	<0.0078	<0.0078	n.t.	n.t.
*P. mirabilis*	>20	>20	>20	>20	>20	>20	>20	>20	<0.15	<0.15	<0.0078	<0.0078	n.t.	n.t.
*P. aeruginosa*	20	>20	20	>20	20	>20	>20	>20	>20	>20	0.5	1	n.t.	n.t.
**Gram-positive bacteria**														
*E. faecalis*	10	>20	10	>20	10	>20	10	>20	<0.15	<0.15	n.t.	n.t.	<0.0078	<0.0078
*L. monocytogenes*	10	>20	20	>20	10	>20	10	>20	<0.15	<0.15	<0.0078	<0.0078	n.t.	n.t.
MRSA	5	>20	10	>20	5	>20	10	>20	<0.15	<0.15	n.t.	n.t.	0.25	0.5

MRSA- Methicillin resistant *Staphylococcus aureus*; MIC: minimal inhibitory concentration; MBC: minimal bactericidal concentration; n.t.: not tested.

**Table 5:** Cytotoxic and anti-inflammatory activities of extracts obtained from the basil samples (mean ± SD, *n* = 3).

Samples	Extracts	Cytotoxic Activity GI_50_ Values (µg/mL)					Anti-Inflammatory Activity EC_50_ (µg/mL)
		NCI H460	MCF7	HeLa	HepG2	PLP2	RAW264.7
*O. basilicum* cv. ’Cinnamon’	Infusion	>400	255 ± 6	271 ± 8	317 ± 6	>400	>400
	EtOH:H_2_O	>400	273 ± 14	310 ± 5	322 ± 6	>400	>400
*O. × citriodorum*	Infusion	>400	281 ± 8	297 ± 16	321 ± 10	>400	>400
	EtOH:H_2_O	161 ± 9	89 ± 4	93 ± 3	114 ± 2	234 ± 21	191 ± 7

GI_50_ values correspond to the sample concentration achieving 50% of growth inhibition in human tumor cell lines or in liver primary culture PLP2. Ellipticine GI_50_ values: 1.21 μg/mL (MCF-7); 1.03 μg/mL (NCI-H460); 0.91 μg/mL (HeLa); 1.10 μg/mL (HepG2), and 2.29 μg/mL (PLP2). Dexamethasone EC_50_ value = 1.6 ± 0.2 μg/mL (RAW294.7).
